# Fluconazole tolerance is associated with altered extracellular vesicle-mediated epithelial immune responses in *Candida albicans*

**DOI:** 10.3389/fmicb.2026.1807232

**Published:** 2026-04-28

**Authors:** Patcharin Thammasit, Artid Amsri, Phimchat Suwannaphong, Yuanji Teng, Huamei Wei, Sirida Youngchim

**Affiliations:** 1 Department of Microbiology, Faculty of Medicine, Chiang Mai University, Chiang Mai, Thailand; 2 Office of Research Administration, Chiang Mai University, Chiang Mai, Thailand; 3 Center for Medical Laboratory Science, Affiliated Hospital of Youjiang Medical University for Nationalities, Baise, Guangxi, China; 4 Clinicopathological Diagnosis and Research Center, Affiliated Hospital of Youjiang Medical University for Nationalities, Baise, Guangxi, China

**Keywords:** *Candida albicans*, extracellular vesicles, fluconazole tolerance, host-pathogen interaction, innate immunity, oral epithelial cells

## Abstract

*Candida albicans* is a major opportunistic fungal pathogen and a leading cause of oropharyngeal candidiasis, particularly in immunocompromised individuals. Extracellular vesicles (EVs) have emerged as important mediators of fungal-host communication; however, whether antifungal tolerance is associated with EV-mediated epithelial immune responses remains poorly understood. Here, we investigated the immunomodulatory effects of EVs released by fluconazole-tolerant and fluconazole-susceptible *C. albicans* clinical isolates on human oral epithelial cells (FaDu). EVs from tolerant and susceptible isolates exhibited comparable size distributions, protein content, cytotoxicity, uptake efficiency, and effects on epithelial barrier integrity. Despite these similarities, EVs from fluconazole-tolerant isolates consistently induced stronger epithelial immune activation, including significantly higher expression of proinflammatory cytokines (*IL-1β*, *IL-6*, *TNF-α*), antimicrobial peptides (*hBD-2* and *hBD-3*), and increased nitric oxide production compared with EVs from susceptible isolates. These differences were reproducible across independent biological replicates and were independent of EV association, cytotoxicity, or barrier disruption. Collectively, these findings indicate that fluconazole tolerance is associated with altered EV-mediated immunomodulatory capacity of *C. albicans* EVs at the epithelial interface within this experimental system. This work provides a functional framework for understanding how tolerant isolates may modulate mucosal immunity and could contribute to mucosal persistence through immune conditioning rather than increased invasive capacity.

## 1 Introduction

Pathogenic fungi have emerged as significant causes of morbidity and mortality among immunocompromised individuals, including patients with HIV/AIDS, cancer receiving chemo- or radiotherapy, organ transplant recipients, and those on long-term immunosuppressive therapy. Oropharyngeal candidiasis (OPC) is one of the most common complications in such patients, particularly following head and neck radiation therapy, and is associated with pain, dysgeusia, anorexia, malnutrition, and risk of esophageal or systemic dissemination (Bensadoun et al., 2011). Clinically, OPC manifests as pseudomembranous lesions (thrush), erythematous candidiasis, or angular cheilitis, and often begins with *Candida* colonization of the oral mucosa (Bensadoun et al., 2011).

Although *Candida* species are commensals of the oral microbiota, they can shift from commensalism to pathogenicity under conditions of immune suppression, epithelial damage, or microbiome imbalance. *Candida albicans* remains the predominant etiological agent of candidiasis worldwide and is responsible for more than 400,000 cases of invasive infection annually, with mortality rates approaching 75% (Brown et al., 2012; Erfaninejad et al., 2022; Suwannaphong et al., 2025). In addition to superficial and systemic infections, *C. albicans* has also been implicated in oral premalignant and malignant disorders (Amer et al., 2017; Gainza-Cirauqui et al., 2013). Its pathogenic success is attributed to multiple virulence and fitness traits, including morphological plasticity, biofilm formation, metabolic adaptability, and immune evasion. Among these traits, the yeast-to-hypha transition plays a central role in epithelial adhesion, tissue invasion, and immune modulation (Almeida et al., 2008; Dalle et al., 2010). Oral epithelial cells constitute both a physical and immunological barrier and actively participate in host defense by sensing fungal components and producing antimicrobial peptides and proinflammatory mediators (Millet et al., 2020; Verma et al., 2017). These epithelial responses are essential for maintaining the balance between fungal commensalism and pathogenicity (Dalle et al., 2010).

Extracellular vesicles (EVs) are lipid bilayer-enclosed structures secreted by both prokaryotic and eukaryotic cells, carrying a diverse array of molecular cargo that reflects the metabolic and environmental state of the producing cell (Rizzo et al., 2020). In bacteria and mammalian systems, EVs are known to mediate intercellular communication (Paolicelli et al., 2019; Rizzo et al., 2020), immunomodulation (Kuipers et al., 2018), and drug resistance (Namee and O'Driscoll, 2018; Xavier et al., 2020). For example, *Acinetobacter baumannii* secretes outer membrane vesicles that mediate horizontal transfer of carbapenemase genes (Jin et al., 2011; Rumbo et al., 2011) and neutralize antimicrobial peptides (Manning and Kuehn, 2011). Similarly, EVs can be internalized by recipient cells, modulating signaling pathways under both physiological and pathological conditions (Yanez-Mo et al., 2015). Although fungal EVs have been less extensively characterized, accumulating evidence indicates that Candida EVs transport virulence-associated proteins, cell wall components, lipids, and RNA species that contribute to adhesion, biofilm formation, immune evasion, and host immune modulation (Karkowska-Kuleta et al., 2020; Kulig et al., 2024). Recent studies further suggest that fungal EVs play critical roles in intraspecies communication and host–pathogen interactions, highlighting their potential as diagnostic and therapeutic targets (Bitencourt et al., 2022; Liu and Hu, 2023).

Fluconazole remains a first-line antifungal agent for OPC; however, increasing clinical failure has been associated with antifungal tolerance, a reversible phenotype characterized by survival at supra-MIC drug concentrations and distinct from stable genetic resistance (Rosenberg et al., 2018; Yang et al., 2023). Antifungal tolerance is defined as a subpopulation-based phenomenon in which a fraction of cells transiently survives antifungal exposure and has been linked to persistent candidemia and treatment failure (Berman and Krysan, 2020; Cohen et al., 2013; Huemer et al., 2020). Despite its growing clinical relevance, the impact of antifungal tolerance on EV-mediated host immune responses remains poorly understood. In particular, whether tolerance shapes the capacity of EVs to modulate epithelial immunity has not been systematically investigated.

Rather than focusing on individual signaling pathways, this study aimed to establish whether fluconazole tolerance is associated with consistent functional differences in EV-mediated epithelial responses at the phenotypic level. Using EVs derived from clinical *C. albicans* isolates obtained from head and neck cancer patients with OPC, we compared the immunomodulatory effects of vesicles from fluconazole-tolerant and fluconazole-susceptible isolates on human oral epithelial cells. We hypothesized that EVs from tolerant isolates exhibit enhanced immunostimulatory properties that promote epithelial innate immune activation. By defining tolerance-associated EV phenotypes, this work provides a foundation for future mechanistic and translational studies on fungal persistence at the oral mucosal surface.

## 2 Materials and methods

### 2.1 Ethics statement

This study was granted exemption from ethical review (Exemption 8270/2565; approval number MIC-2564-08270) by the Research Ethics Committee, Faculty of Medicine, Chiang Mai University, and was approved for biosafety research from the Institutional Biosafety Committee (IBC), Faculty of Medicine, Chiang Mai University (Study code CMUIBC0266018).

### 2.2 *Candida albicans* strains and growth conditions

Clinical isolates of *C. albicans* were obtained from head and neck cancer patients with oral candidiasis and their antifungal susceptibility profiles were characterized as previously described (Suwannaphong et al., 2025; Ueangphairot et al., 2025). Four isolates were selected for this study: two fluconazole-tolerant isolates and two fluconazole-susceptible isolates as listed in Table 1. Reference strains *C. albicans* ATCC90028 and SC5314 are well-characterized fluconazole-susceptible laboratory strains and were included as technical controls for EV isolation, physicochemical characterization, and cytotoxicity assessment. All strains were routinely cultured in Sabouraud Dextrose Broth (SDB) at 37 °C with shaking at 180 rpm.

**Table 1:** *Candida albicans* strains used in this study.

Strain	Classification*	Designation	Characteristic	Reference
*C. albicans* L1	Moderately tolerant	T1	Clinical isolate	Suwannaphong et al. (2025)
*C. albicans* L6	Highly tolerant	T2	Clinical isolate	This study
*C. albicans* A3	Susceptible/Non-tolerant	S1	Clinical isolate	Suwannaphong et al. (2025)
*C. albicans* D2	Susceptible/Non-tolerant	S2	Clinical isolate	This study
*C. albicans* ATCC90028	Susceptible	–	Reference strain	–
*C. albicans* SC5314	Susceptible	–	Reference strain	–

*Classification was defined by drug susceptibility (MIC-based, CLSI/EUCAST standards) and Supra-MIC Growth (SMG) values.

### 2.3 Isolation and characterization of EVs

#### 2.3.1 EV isolation by ultracentrifugation

EVs were isolated as described by Gil-Bona et al. (2015a) and Martinez-Lopez et al. (2022), with minor modifications. Briefly, *C. albicans* cells were adjusted to 1 × 10^6^ cells/mL and cultured in 200 mL of yeast nitrogen base with sucrose (YNBS; 5 g/L ammonium sulfate, 1.7 g/L yeast nitrogen base, 20 g/L sucrose, and 75 mM tartaric acid, pH 4.0) for 16 h at 37 °C, 180 rpm. Cell-free supernatants were obtained by centrifugation at 8,000 rpm for 20 min at 4 °C, followed by filtration through a 0.45 μm membrane. The fungal cell-free supernatants were concentrated using Amicon Ultra-15 centrifugal filters (100-kDa cutoff; Millipore, MA, USA) to a final volume of 8 mL and then ultracentrifuged at 150,000 × g for 1 h by Optima MAX-XP ultracentrifuge (Beckman Coulter, Brea, CA, USA). The resulting EV pellets were washed twice with sterile PBS (pH 7.4) and resuspended for downstream analyses. All procedures were performed at 4 °C to preserve vesicle integrity. Isolated EVs were stored at −80 °C and thawed on ice only once prior to functional assays to minimize freeze–thaw-induced vesicle disruption.

#### 2.3.2 EV size determination by dynamic light scattering (DLS)

EV size distribution (Z-average diameter) and polydispersity were measured by dynamic light scattering (DLS) using a Zetasizer Nano ZS (Malvern, UK). EV samples from fluconazole-tolerant and -susceptible isolates were diluted 1:100 in 0.22 μm-filtered PBS to achieve optimal scattering intensity. Each sample was measured in ten technical replicates, and the Z-average diameter and polydispersity index (PDI) were recorded using Zetasizer software. DLS measurements were performed for EV physicochemical characterization, and size distributions were reported descriptively.

#### 2.3.3 EV protein quantification

For lysis, EV suspensions in PBS were subjected to sonication at 20% amplitude for 10 s (3 cycles, with 30-s intervals on ice), followed by three freeze–thaw cycles (−80 °C to room temperature) to ensure complete vesicle disruption. Total protein concentration was determined using bicinchoninic acid (BCA) assay (Pierce BCA Protein Assay, Thermo Scientific, Rockford, IL, USA) according to the manufacturer’s instructions. Briefly, 25 μL of samples or bovine serum albumin (BSA) standard series (0–2,000 μg/mL) were mixed with 200 μL of working reagent (reagents A and B, 50:1 ratio) and incubated at 37 °C for 30 min. Absorbance was measured at 562 nm using a spectrophotometer, and protein concentrations were calculated from the BSA standard curve. EV dosing in subsequent functional assays was normalized based on total protein content, which was used as a proxy for relative vesicle abundance; however, this approach does not account for potential differences in particle number or cargo composition across isolates. This protocol was adapted from established methods for extracellular vesicle protein analysis (Stec et al., 2022). All measurements were performed using three independent EV preparations per isolate (*n* = 3), and results are presented as mean ± standard deviation.

### 2.4 FaDu oral epithelial cell culture

FaDu oral epithelial cells (derived from human hypopharyngeal carcinoma) were kindly provided by Assistant Professor Dr. Jiraporn Kantapan, Faculty of Associated Medical Sciences, Chiang Mai University. Cells were maintained in Dulbecco’s Modified Eagle Medium (DMEM; Gibco, MA, USA) supplemented with 10% fetal bovine serum (FBS; Gibco, MA, USA), 500 U/mL penicillin, and 500 μg/mL streptomycin at 37 °C in a 5% CO₂ humidified incubator. For experiments, confluent monolayers were serum-starved overnight in serum-free DMEM.

### 2.5 Cytotoxicity and epithelial barrier integrity

#### 2.5.1 MTT assay

FaDu cells (5 × 10^3^ cells/well) were seeded in 96-well plates and incubated for 16 h at 37 °C in a 5% CO₂ humidified incubator. EVs were applied at protein concentrations of 5, 10, or 20 μg/mL for 8 h or 24 h. Untreated cells served as negative controls, and cells treated with 0.1% Triton X-100 were used as positive controls.

After treatment, 20 μL of MTT solution (5 mg/mL) were added per well and incubated for 4 h. The supernatant was removed, and 200 μL of dimethyl sulfoxide (DMSO) were added to solubilize formazan crystals. Absorbance was read at 570 nm using a microplate reader (BioTek™ Synergy H4 Hybrid Microplate Reader, VT, USA). Cell viability (%) was calculated relative to untreated controls. Experiments were performed in triplicate wells with three independent biological replicates.

#### 2.5.2 Lactate dehydrogenase (LDH) release assay

Cytotoxicity was further evaluated using the CyQUANT™ LDH Cytotoxicity Assay Kit (Thermo Fisher Scientific, MA, USA) according to the manufacturer’s instructions. Culture supernatants were collected at 8, 24, and 48 h post-EV treatment (20 μg/mL protein). Briefly, 50 μL of sample or spontaneous LDH release (low control) or maximum LDH release (high control) was transferred to a 96-well plate and mixed with 50 μL of reaction mixture. Plates were incubated for 30 min at room temperature, protected from light. Absorbance was measured at 490 nm with reference to 680 nm using a microplate reader. Measurements were performed in technical triplicate and biological triplicate. Cell viability was calculated from LDH measurements and expressed as percentage of survival relative to the untreated control, which was set to 100%.

#### 2.5.3 *Trans*-epithelial electrical resistance (TEER) measurement

FaDu cells were seeded on collagen type I-coated Transwell inserts (polycarbonate membrane, 0.4 μm pore size, 12 mm diameter; Corning Costar) at a density of 1 × 10^5^ cells/insert and maintained in complete medium until confluent monolayers with stable baseline TEER values were achieved (typically 5–7 days). EVs were applied to the apical chamber at a protein concentration of 20 μg/mL. TEER was measured from day 1 to day 5 using an EVOM2 volt-ohmmeter (World Precision Instruments, Sarasota, FL, USA). Background resistance from cell-free inserts was subtracted, and TEER value (*Ω*·cm^2^) was calculated as:


$$\text{TEER value}\;(\Omega \cdot {\mathrm{cm}}^{2})=(R\;\text{sample}-R\;\text{blank})\times \text{membrane area}$$

where R is resistance (Ω) and the effective membrane area of 12 mm diameter is 1.12 cm^2^. TEER values are presented as absolute values relative to baseline (time 0).

### 2.6 Uptake and cellular association of EVs by flow cytometry and fluorescence microscopy

EV uptake was assessed using fluorescein isothiocyanate (FITC)–labeled EVs. EVs were labeled with 50 μg/mL of FITC (Sigma-Aldrich, St. Louis, MO, USA) in 0.1 M carbonate buffer (pH 9.0) overnight at 4 °C with gentle shaking, followed by quenching with 10% BSA. Labeled EVs were washed three times with filtered PBS and stored at 4 °C in the dark until use.

FaDu cells (1 × 10^5^ cells/well) were seeded in 24-well plates and incubated overnight in serum-free DMEM. Cells were then exposed to FITC-labeled EVs at protein concentrations of 20 μg/mL for 8 h or 24 h at 37 °C in 5% CO₂. After incubation, cells were washed three times with PBS, detached with 0.25% trypsin–EDTA, and resuspended in PBS containing 1% BSA. EV-associated fluorescence was quantified by flow cytometry (DxFLEX Flow Cytometer, Beckman Coulter Inc., CA, USA) and visualized using fluorescence microscopy. FITC-positive cells were interpreted as EV-associated cells and unlabeled EVs and untreated cells were included as negative controls.

### 2.7 Immune response assays

#### 2.7.1 Cytokine and antimicrobial peptide expression by qRT-PCR

FaDu cells were treated with EVs at protein concentrations of 20 μg/mL for 24 h. Lipopolysaccharide (LPS; 100 ng/mL) was used as positive control to verify epithelial immune responsiveness and assay performance in cytokine, antimicrobial peptide analyses. Total RNA was extracted using NucleoSpin RNA kit (Macherey and Nagel, Germany) according to the manufacturer’s instructions and RNA purity was assessed based on 260/280 nm absorbance ratio. Equal amounts of RNA was reverse transcribed using the Maxime™ RT PreMix Kit (Oligo (dT)_15_ Primer, iNtRON Biotechnology, Korea) in a final volume of 20 μL. After that, real-time RT-PCR assays were carried out using SensiFAST SYBR No-ROX Kit (Bioline, London, UK) in a final volume of 20 μL on an ABI 7500 Real-Time Detection System (Applied Biosystems, Waltham, MA, USA). Relative expression levels were calculated using the 2^−∆∆Ct^ method, normalized to a housekeeping gene *β*-actin. All experiments were performed in three independent replicates. Primer sequences used for real-time PCR are provided in Supplementary Table S1.

#### 2.7.2 Cytokine measurement

The culture supernatants were collected and stored at −20 °C until the cytokine assay was performed. IL-6 concentrations were measured using a sandwich enzyme linked immunosorbent assay (ELISA) kit according to the manufacturer’s instructions (BioLegend, San Diego, CA, USA). Measurements were performed in three biological replicates.

#### 2.7.3 Nitric oxide (NO) production

Nitric oxide (NO) production was quantified using the Griess reagent assay (Kleinbongard et al., 2002), which measures nitrite, a stable oxidation product of NO. FaDu cells were treated with different *C. albicans* EVs at protein concentrations of 20 μg/mL at 3 and 6 h. Briefly, 100 μL of culture supernatants were mixed with an equal volume of Griess reagent and incubated for 30 min at room temperature. Absorbance was measured at 540 nm, and nitrite concentrations were calculated from a sodium nitrite standard curve. LPS (5 μg/mL) and untreated cells served as positive and negative controls, respectively. All measurements were performed in three biological replicates.

### 2.8 Statistical analysis

Experiments were performed using at least three independent biological replicates. Each biological replicate represents an independent EV preparation derived from an independent *C. albicans* culture for each isolate. Data are expressed as mean ± standard deviation (SD). Statistical analyses were conducted using GraphPad Prism version 10.6.1. Two-group comparisons were performed using unpaired Student’s *t*-tests and multi-group comparisons were conducted using one-way or two-way ANOVA with Tukey’s *post hoc* test. For immune-response analyses, isolates were grouped as fluconazole-tolerant or -susceptible. A *p*-value <0.05 was considered statistically significant. For analysis involving grouped comparisons (fluconazole-tolerant vs. -susceptible), isolates were treated as representative of their respective phenotypic categories. However, given the limited number of isolates per group, these analyses are intended to identify phenotype-associated trends rather than to support definitive population-level inference.

## 3 Results

### 3.1 Characterization of EVs from fluconazole-tolerant and -susceptible *C. albicans*

**Figure 1:**
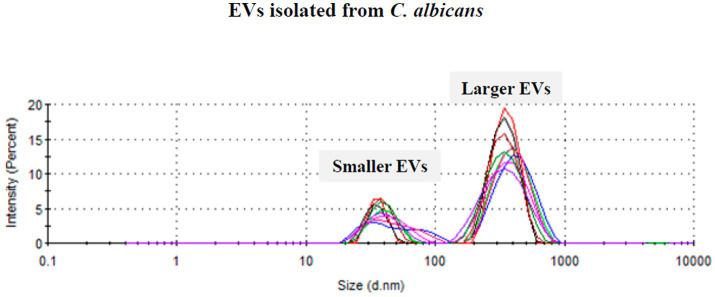
Representative size distribution of EVs isolated from a single *C. albicans* isolate measured by dynamic light scattering (DLS). The DLS profile illustrates two distinct EV populations: smaller vesicles (20–60 nm) and larger vesicles (200–500 nm), with the larger population being predominant. Colors indicate 10 consecutive measurements for each displayed isolate, with values presented as the mean.

EVs were successfully isolated from fluconazole-tolerant (T1, T2), fluconazole-susceptible (S1, S2), and reference *C. albicans* strains (ATCC90028 and SC5314). Dynamic light scattering (DLS) analysis consistently demonstrated the presence of two distinct EV populations in all isolates: a smaller population with diameters ranging from 20 to 60 nm and a larger population ranging from 200 to 500 nm, with the larger population being predominant in all isolates (Figure 1 and Table 2). All isolates exhibited both EV populations with broadly similar size distributions. At the descriptive level, EVs from fluconazole-tolerant isolates appeared to show a relative enrichment of larger vesicles compared with susceptible isolates, while a similar distribution was observed for the reference strain ATCC90028. The polydispersity index (PDI) values reflected moderately homogeneous preparations, indicating that the EV isolation protocol yielded reproducible and well-defined vesicle populations across isolates. Importantly, these physicochemical analyses were descriptive and were not used to infer functional differences between EV subpopulations, as all downstream assays were performed using total EV preparations to reflect physiologically relevant epithelial exposure.

**Table 2:** Particle size distribution and polydispersity index (PDI) of EVs isolated from *C. albicans* strains measured by DLS.

Designation of *C. albicans* isolate		Smaller EVs			Larger EVs		
		Size (nm)	%	PDI	Size (nm)	%	PDI
T1	Moderately tolerant	31.53 ± 5.34	25.4	0.28	357.89 ± 4.82	74.6	0.31
T2	Highly tolerant	53.01 ± 2.76	11.9	0.32	423.49 ± 7.51	88.1	0.29
S1	Susceptible	31.42 ± 10.84	24.8	0.27	292.02 ± 11.17	75.2	0.30
S2	Susceptible	55.70 ± 4.10	27.5	0.31	324.09 ± 3.42	72.5	0.28
ATCC 90,028	Reference	26.36 ± 8.12	12.9	0.26	233.81 ± 9.74	87.1	0.30
SC5314	Reference	57.81 ± 2.68	17.6	0.30	424.47 ± 12.14	82.4	0.32

Data represent mean ± SD of 10 technical measurements per EV preparation.

The total protein content of each 400 μL EV suspension was quantified and used as an indirect indicator of vesicle abundance. Total protein levels ranged from 16 to 23 μg across all isolates (Table 3). Fluconazole-tolerant isolates exhibited a trend toward higher protein content compared with susceptible isolates; however, variability across replicates resulted in overlapping values between groups. These findings are consistent with broadly comparable EV yields across isolates and support the use of protein-based normalization for downstream functional assays.

**Table 3:** Total protein content of EVs isolated from *C. albicans* isolates.

Designation of *C. albicans* isolate		Protein content (μg/400 μL EV suspension, mean ± SD)
T1	Moderately tolerant	21.68 ± 3.21
T2	Highly tolerant	23.14 ± 2.64
S1	Susceptible	17.82 ± 1.51
S2	Susceptible	19.54 ± 1.65
ATCC90028	Reference	21.64 ± 1.97
SC5314	Reference	16.74 ± 2.78

Values represent mean ± SD from *n* = 3 independent EV preparations per isolate. Protein content was used for normalization of EV input in downstream functional assays.

### 3.2 Assessment of EV-induced cytotoxicity in FaDu oral epithelial cells

**Figure 2:**
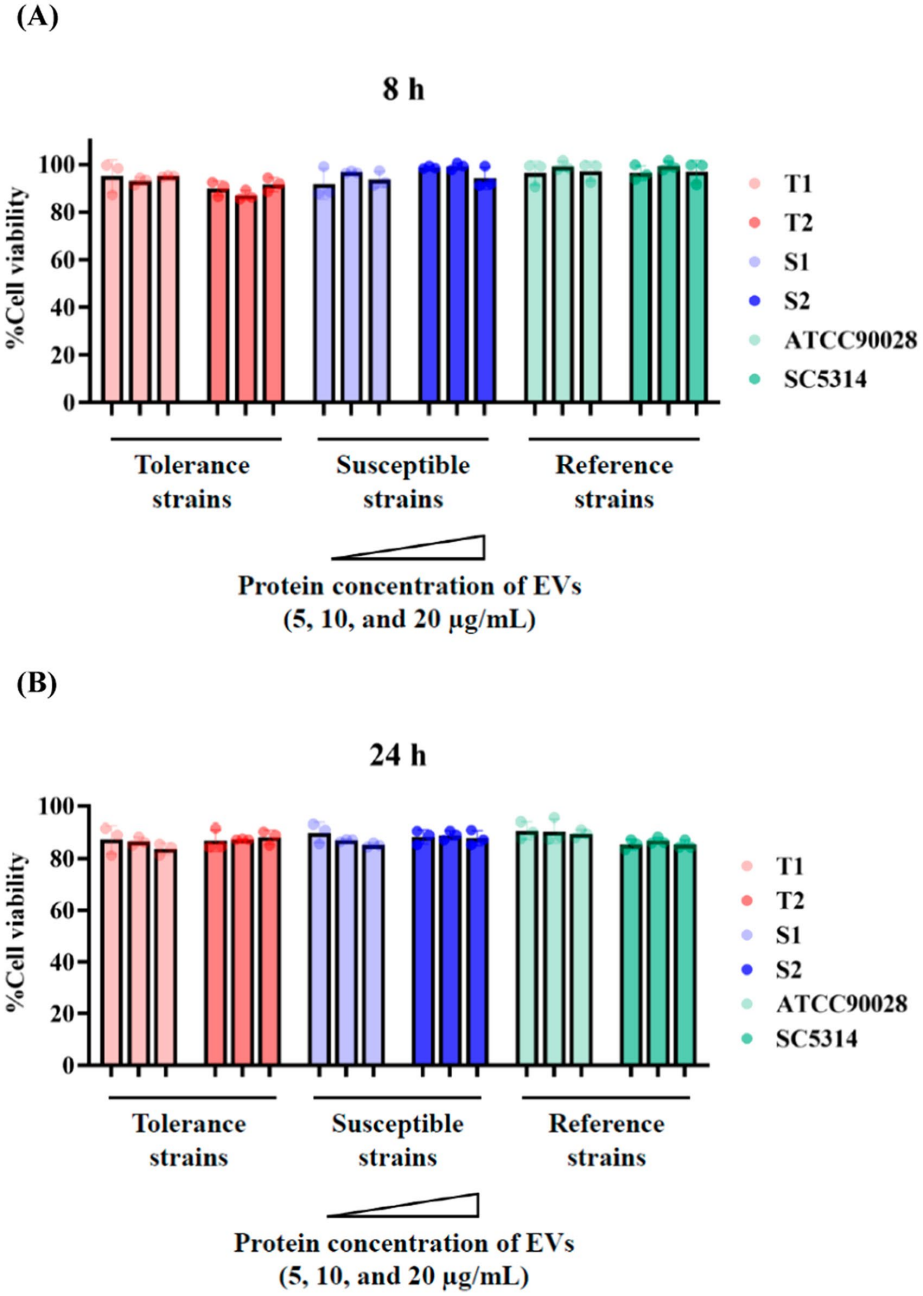
Cytotoxicity of *C. albicans* EVs on FaDu oral epithelial cells. Cell viability was assessed using MTT assay after **(A)** 8 h and **(B)** 24 h of exposure to EVs derived from fluconazole-tolerant (T1, T2), fluconazole-susceptible (S1, S2), and reference strains (ATCC90028, SC5314) at protein concentrations of 5, 10, and 20 μg/mL. Data are presented as mean ± SD of three independent experiments.

The cytotoxic effects of *C. albicans* EVs toward FaDu epithelial cells were evaluated using the MTT and lactate dehydrogenase (LDH) release assays. For the MTT assay, cells were exposed to EVs at protein concentrations of 5, 10, and 20 μg/mL for 8 and 24 h. As illustrated in Figures 2A,B, cell viability remained above 85% across all experimental groups and time points. Because individual clinical isolates exhibit variable baseline phenotypes, immune-response data were analyzed at the group level (fluconazole-tolerant vs. fluconazole-susceptible) to identify tolerance-associated EV effects. Given the limited number of isolates per group, these comparisons should be interpreted as indicative of phenotype-associated trends within this dataset. No statistically significant differences in cell viability were observed between EVs from fluconazole-tolerant and fluconazole-susceptible isolates or between EV-treated and untreated controls. These findings indicate that *C. albicans* EVs, even at the highest concentrations tested, exhibit minimal cytotoxicity toward oral epithelial cells.

**Figure 3:**
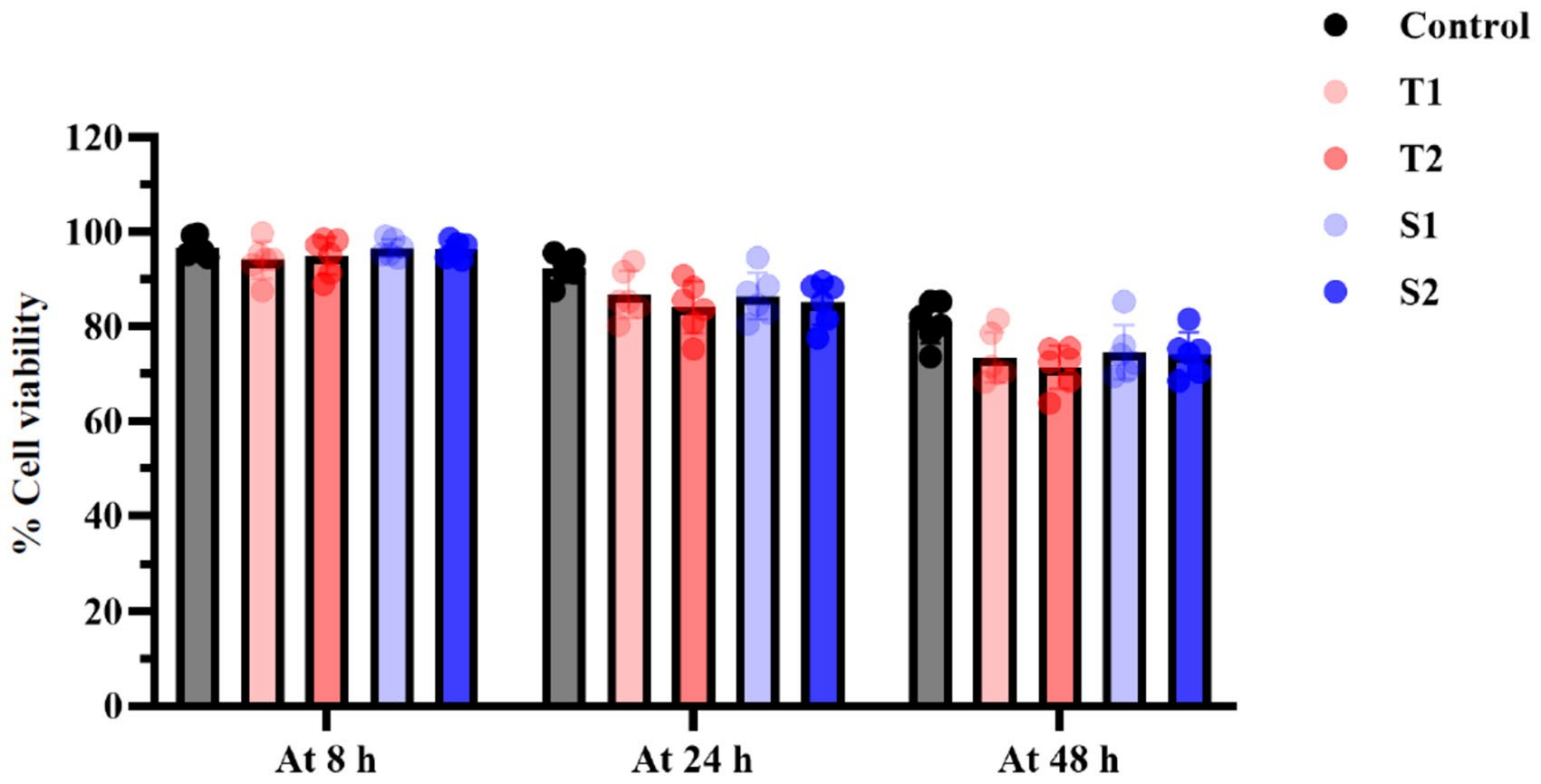
LDH-based assessment of epithelial membrane integrity following EV exposure. LDH activity was measured in culture supernatants at 8, 24, and 48 h post-treatment with 20 μg/mL EVs. Untreated cells were used as the control and set to 100% survival. Data represent mean ± SD of three independent experiments.

To further assess epithelial membrane integrity, LDH release was measured at 8, 24, and 48 h following exposure to EVs (20 μg/mL). Consistent with the MTT results, EV treatment did not induce significant LDH release compared with untreated controls at any time point (Figure 3). Although minor variations were observed among individual isolates, these differences were not statistically significant when analyzed at the group level. Collectively, these complementary assays demonstrate that *C. albicans* EVs do not induce cytotoxicity or membrane damage in FaDu cells under the experimental conditions used. This confirms that the observed EV-mediated immune responses occur independently of epithelial cell death and supports the suitability of this *in vitro* model for subsequent immunological analyses.

### 3.3 Effects of EVs on epithelial barrier integrity

**Figure 4:**
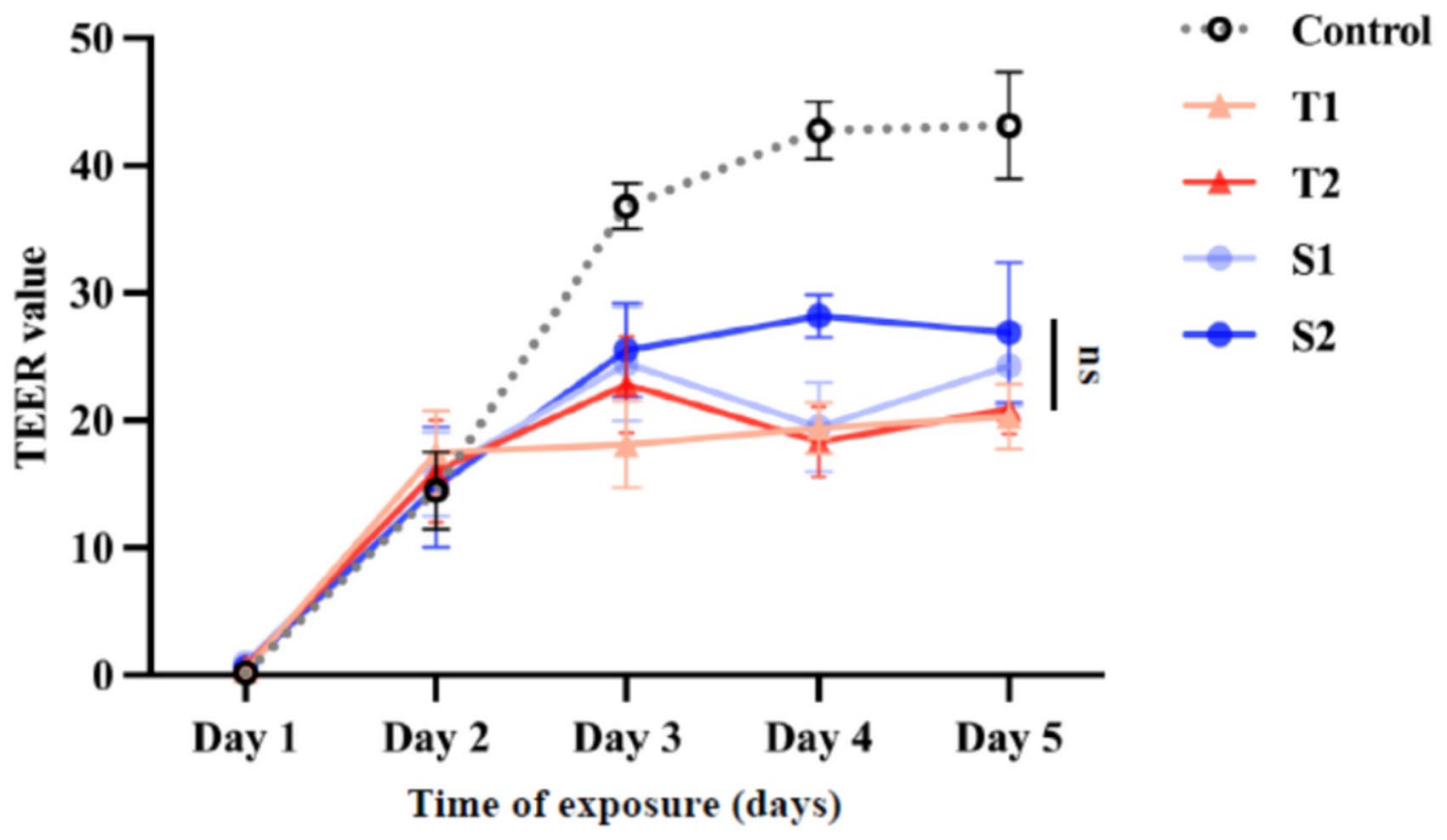
Transepithelial electrical resistance (TEER) of FaDu monolayers after EV treatment. TEER was monitored over 5 days after treatment with 20 μg/mL EVs. Values represent mean ± SD of three independent experiments.

To determine whether EVs affect epithelial barrier integrity, TEER values were monitored over five days following EV exposure. EV-treated monolayers exhibited a modest reduction in TEER compared with untreated controls at early time points, followed by partial recovery over time (Figure 4). When analyzed at the group level, no statistically significant differences were observed between EVs derived from fluconazole-tolerant and fluconazole-susceptible isolates at any time points. TEER profiles of individual isolates were largely overlapping, including comparable responses between representative tolerance and susceptible isolates.

These results indicate that exposure to *C. albicans* EVs induces a transient and reversible modulation of epithelial barrier properties without causing sustained barrier disruption. The absence of significant differences between tolerant and susceptible groups suggests that barrier modulation may represent a general response to EV exposure rather than a tolerance-specific effect.

### 3.4 Uptake and cellular association of EVs by FaDu oral epithelial cells

**Figure 5:**
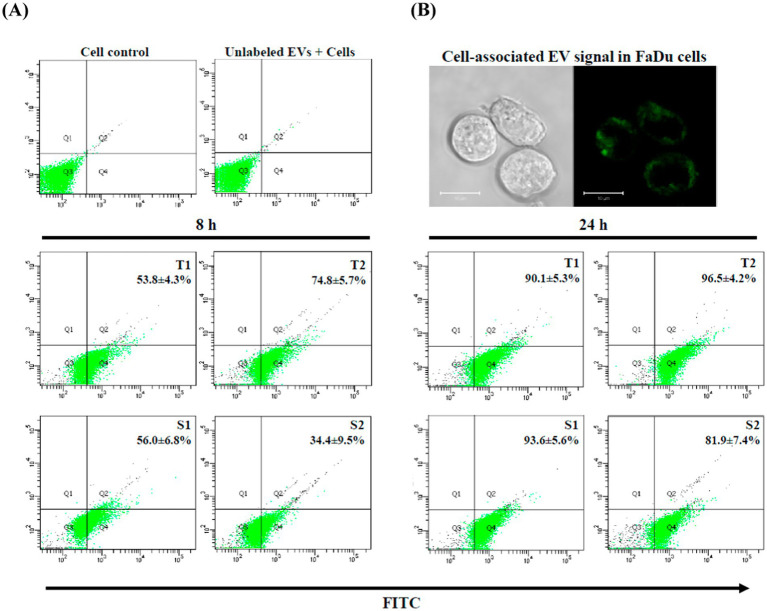
Uptake and cellular association of FITC-labeled EVs by FaDu oral epithelial cells. **(A)** Representative flow cytometry plots showing uptake of FITC-labeled EVs by FaDu cells after 8 h and 24 h exposure to 20 μg/mL EVs derived from fluconazole-tolerant (T1, T2) and fluconazole-susceptible (S1, S2) isolates. EV-positive cells were defined as FITC-positive events (Q2 + Q4) relative to untreated cells and cells treated with unlabeled EVs. Data represent mean ± SD of three independent biological experiments. **(B)** Representative fluorescence microscopy images showing cell-associated FITC signal (green) in FaDu cells after 24 h of EV exposure. Fluorescence is observed in cytoplasmic regions and at the cell periphery, consistent with EV association. Scale bar = 10 μm.

The uptake of FITC-labeled EVs by FaDu oral epithelial cells was quantified using flow cytometry. EV association with cells was efficient and time-dependent across all isolates tested. After 8 h of exposure, approximately 30–75% of cells were FITC-positive, increasing to 80–95% at 24 h (Figure 5A). When analyzed at the group level, no statistically significant differences were observed between EVs derived from fluconazole-tolerant and fluconazole-susceptible isolates at either time point.

Fluorescence microscopy further demonstrated strong cell-associated FITC signal following 24 h of EV exposure, with fluorescence detected in both the cytoplasmic region and at the cell periphery (Figure 5B), consistent with flow cytometry results. Together, these data demonstrate that EV association with epithelial cells is robust and comparable across isolates, suggesting that strain-dependent differences in epithelial immune activation are not attributable to differences in EV uptake efficiency. However, this interpretation is based on cell-associated fluorescence and does not distinguish between surface-bound and internalized EVs.

### 3.5 Immunomodulatory effects of *C. albicans* EVs on FaDu oral epithelial cells

To investigate the immunological consequences of EV exposure at the functional and phenotypic level, cytokine expression, antimicrobial peptide expression, and NO production were examined in EV-treated FaDu cells. Rather than focusing on individual signaling pathways, this analysis was designed to determine whether fluconazole tolerance is associated with reproducible differences in EV-mediated epithelial responses across multiple immune endpoints. For all immune-response analyses, isolates were grouped as fluconazole-tolerant or fluconazole-susceptible, and statistical comparisons were performed at the group level, as defined in the Methods section. All statistical analyses were conducted using raw experimental values (mean ± SD, *n* = 3 independent experiments).

#### 3.5.1 Cytokine expression and IL-6 secretion

**Figure 6:**
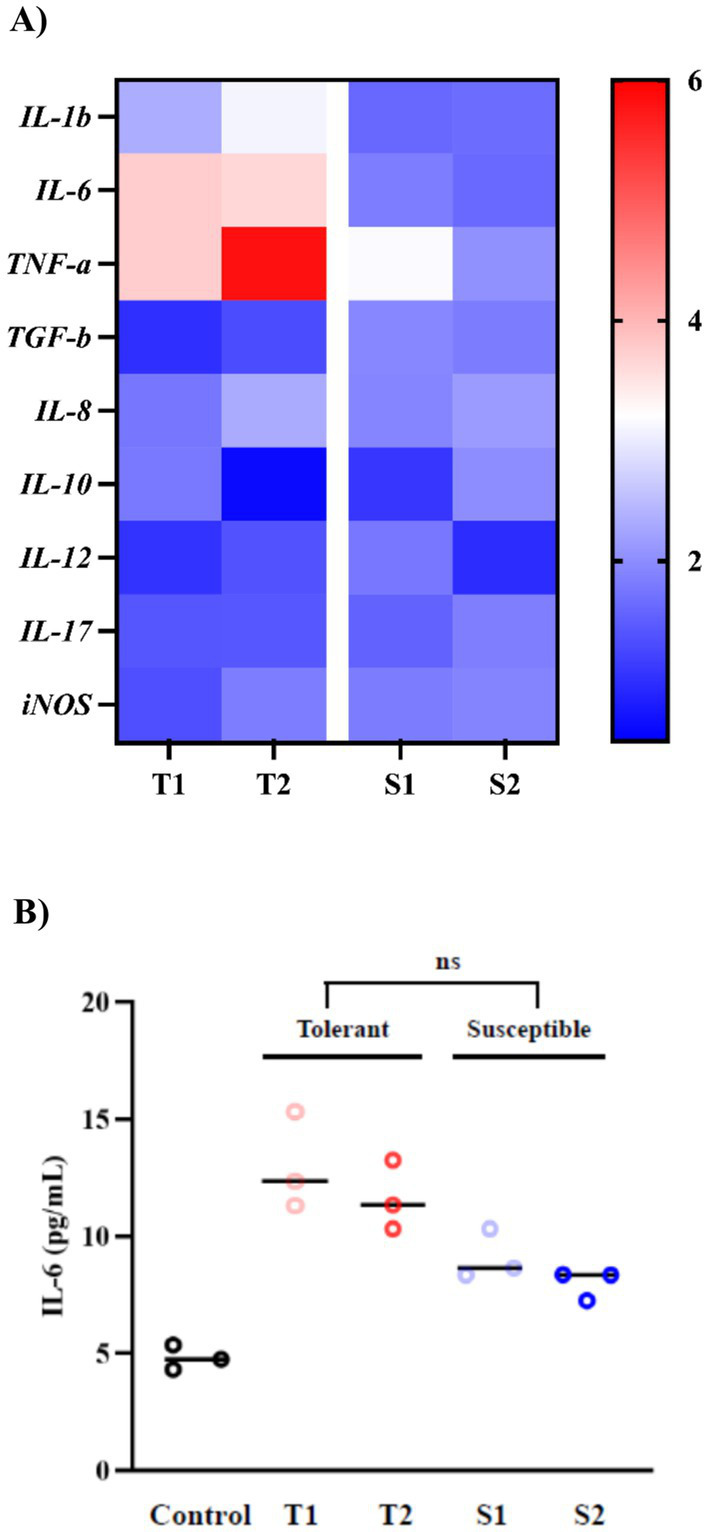
Cytokine responses of FaDu oral epithelial cells to *C. albicans* EVs. **(A)** Relative mRNA expression of nine cytokines measured by qRT-PCR after 24 h treatment with 20 μg/mL EVs. Heat maps show mean ΔΔCt values (*n* = 3 independent experiments) normalized to untreated controls and are presented for visualization of expression patterns. Statistical analyses were performed using raw ΔΔCt values (mean ± SD) at the group level (fluconazole-tolerant vs. fluconazole-susceptible isolates); full raw data and exact *p* values are provided in Supplementary Table S2. **(B)** IL-6 secretion measured by ELISA in culture supernatants after 24 h EV exposure. Statistical comparisons were performed using raw ELISA values at the group level (*p* = 0.065). * indicates a statistically significant difference (*p* < 0.05).

qRT-PCR analysis of FaDu cells treated with 20 μg/mL EVs for 24 h showed distinct patterns of nine cytokines: *IL-1β, IL-6, TNF-α, TGF-β, IL-8, IL-10, IL-12, IL-17*, and *iNOS* (Figure 6A). EVs from both fluconazole-tolerant and fluconazole-susceptible isolates induced overall upregulation of these cytokines relative to untreated controls, indicating that *C. albicans* EVs broadly stimulate epithelial immune responses. When analyzed at the group level using raw ΔΔCt values, EVs from fluconazole-tolerant isolates induced significantly higher expression of *IL-1β, IL-6*, and *TNF-α* compared with EVs from susceptible isolates (*p* < 0.05), whereas other cytokines showed comparable trends without reaching statistical significance. Heat maps depict mean ΔΔCt values to illustrate relative expression patterns across cytokines and strain groups, while full raw data, variance, and exact *p* values are provided in Supplementary Table S2.

Consistent with transcriptional data, IL-6 protein secretion measured by ELISA was higher in FaDu cells treated with EVs from tolerant isolates than those treated with susceptible-isolate EVs, showing a trend toward higher levels at the group level (Figure 6B). LPS-treated cells were included as positive control to verify assay performance and confirm that FaDu cells retained intact immune responsiveness under experimental conditions. This confirms that transcriptional differences observed at the group level translate into functional cytokine production.

#### 3.5.2 Antimicrobial peptide expression

**Figure 7:**
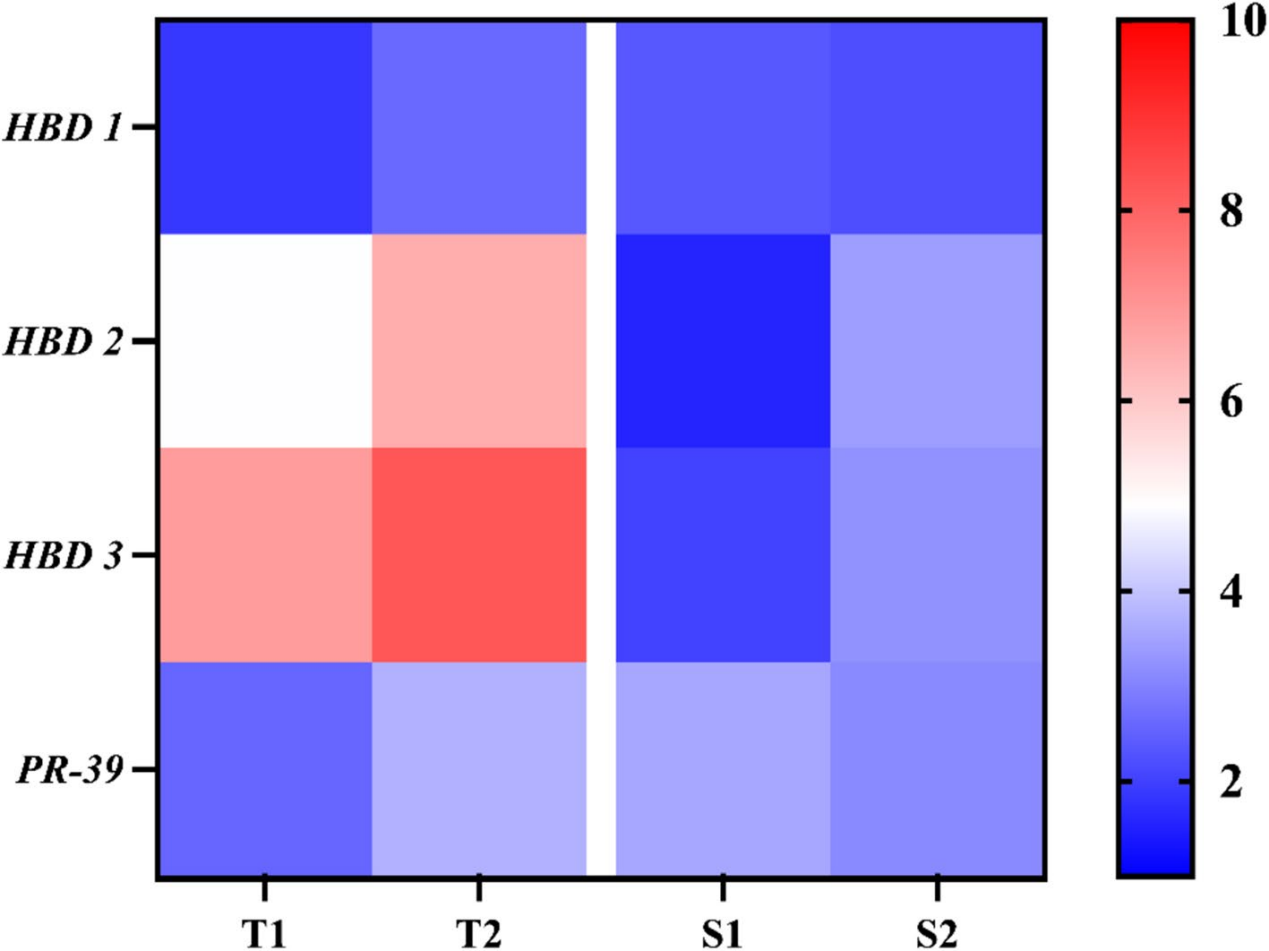
Antimicrobial peptide expression in FaDu cells following exposure to *C. albicans* EVs. Relative expressions of *hBD-1*, *hBD-2*, *hBD-3*, and *PR-39* quantified by qRT-PCR following 24 h exposure to 20 μg/mL EVs. Heat maps display mean ΔΔCt values (*n* = 3 independent experiments) normalized to untreated controls and are shown for visualization purposes only. Statistical comparisons were performed at the group level (fluconazole-tolerant vs. fluconazole-susceptible isolates) using raw experimental values (mean ± SD, *n* = 3). Full raw data, variance, and exact p values are provided in Supplementary Table S3.

To evaluate epithelial antimicrobial defenses, the expression of human β-defensins (*hBD-1, hBD-2, hBD-3*) and porcine cathelicidin (*PR-39*) was examined following EV exposure (Figure 7). EVs from all isolates modestly increased *hBD-1* and *PR-39* expression, with no significant differences observed between tolerant and susceptible groups.

In contrast, EVs from fluconazole-tolerant isolates induced significantly higher expression of *hBD-2* and *hBD-3* compared with EVs from susceptible isolates when analyzed using raw qRT-PCR values at the group level (*p* < 0.05). Heat maps are shown to visualize relative expression patterns, while statistical analyses were performed on raw replicate data (mean ± SD, *n* = 3), with complete results provided in Supplementary Table S3. These findings indicate a selective enhancement of epithelial antimicrobial peptide responses by tolerant-isolate EVs.

#### 3.5.3 Nitric oxide (NO) production

**Figure 8:**
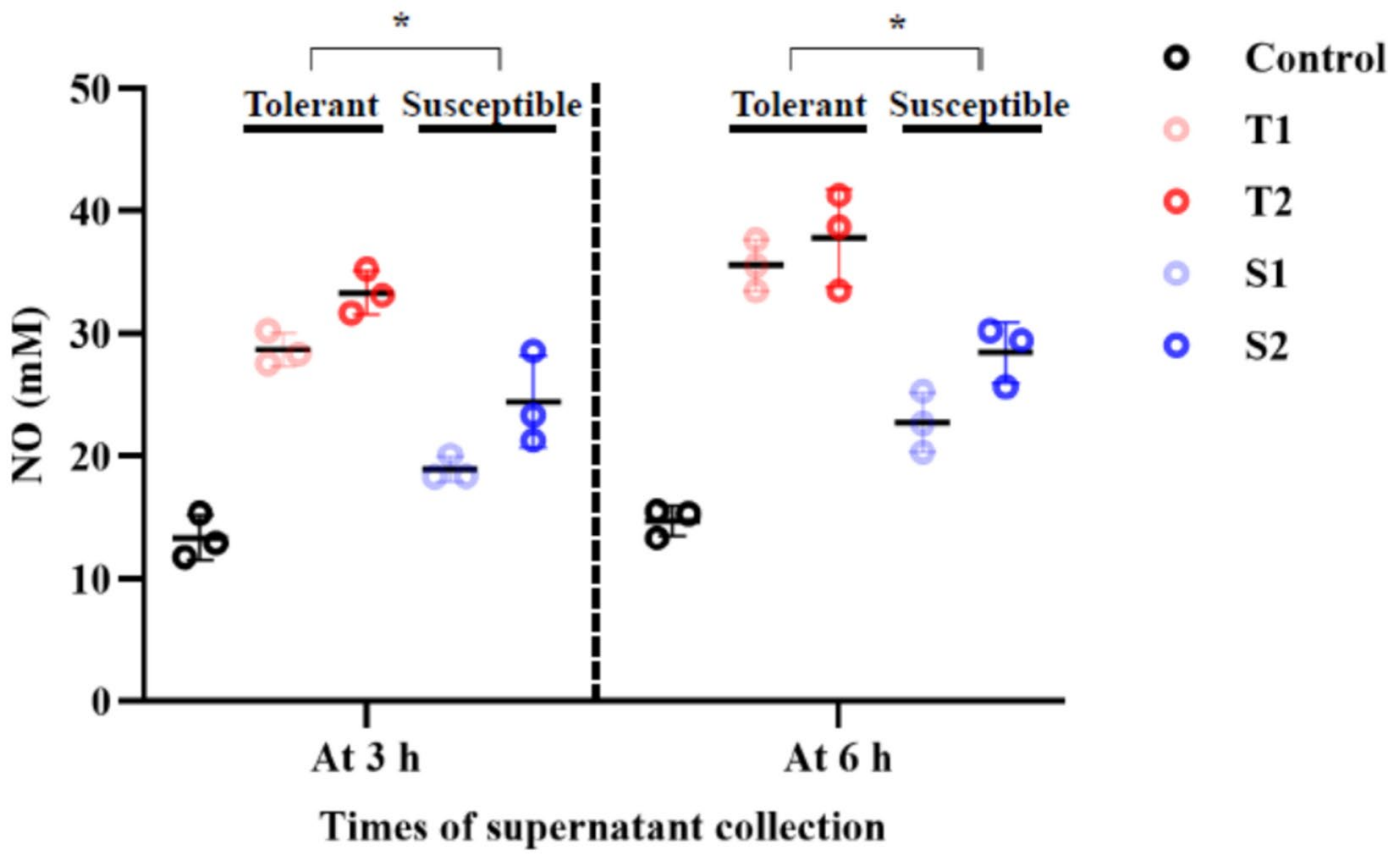
Nitric oxide (NO) production in FaDu cells following EV treatment. NO levels were measured in culture supernatants at 3 h and 6 h post-treatment with 20 μg/mL EVs (mean ± SD, *n* = 3; Supplementary Table S4). One-way ANOVA with Tukey’s *post hoc* test showed significantly higher NO production in cells treated with EVs from fluconazole-tolerant isolates compared with susceptible isolates at 3 h (*p* = 0.0008) and 6 h (*p* = 0.0002). * indicates a statistically significant difference (*p* < 0.05).

NO production was measured in culture supernatants at 3 h and 6 h following EV treatment as a functional marker of epithelial antimicrobial activation (Figure 8). NO levels increased over time in all EV-treated groups, indicating a time-dependent epithelial response to EV exposure.

When analyzed at the group level using raw experimental values, EVs from fluconazole-tolerant isolates stimulated significantly higher NO production at both 3 h and 6 h compared with EVs from susceptible isolates (*p* < 0.05). Full raw data, variance, and statistical comparisons are provided in Supplementary Table S4. These results further support enhanced activation of epithelial innate defenses by EVs derived from tolerant isolates.

Despite similar physicochemical characteristics, cytotoxicity profiles, uptake efficiency, and barrier effects, EVs from the tested fluconazole-tolerant *C. albicans* isolates were consistently associated with stronger epithelial cytokine, antimicrobial peptide, and nitric oxide responses than EVs from susceptible isolates when analyzed at the group level. These differences were reproducible across independent biological replicates and multiple functional endpoints within this dataset, indicating that tolerance-associated EVs are associated with enhanced epithelial immune activation independently of EV uptake, cytotoxicity, or barrier disruption.

## 4 Discussion

In this study, we show that EVs released by the tested fluconazole-tolerant *C. albicans* isolates are associated with stronger immunomodulatory responses in oral epithelial cells than EVs from fluconazole-susceptible isolates. By combining physicochemical characterization, uptake analysis, barrier integrity measurements, and functional immune assays, our findings provide a functional, hypothesis-generating framework for understanding how antifungal tolerance is associated with EV-mediated host-pathogen communication at the oral mucosal surface. Collectively, our results indicate that fungal EVs may contribute to epithelial immune conditioning and suggest a potential, phenotype-associated mechanism by which fluconazole-tolerant *C. albicans* isolates may contribute to mucosal persistence through immune modulation rather than increased invasive capacity.

Fluconazole tolerance is associated with adaptive remodeling of ergosterol biosynthesis, plasma membrane composition, and cellular stress-response pathways (LaFayette et al., 2010; Song et al., 2020). These adaptive processes are expected to influence EV biogenesis, release, and cargo composition. Consistent with this, our findings suggest that the observed differences represent tolerance-associated phenotypic traits within the tested isolates, rather than a transient response to acute drug exposure. Although tolerance has been linked to persistent colonization and treatment failure in clinical settings (Rosenberg et al., 2018), it is not consistently associated with enhanced tissue invasiveness. These findings further support a role for EV-mediated modulation of epithelial immunity in persistence, although this remains hypothesis-generating and requires validation in broader isolate collections and *in vivo* systems.

Consistent with previous reports, *C. albicans* EVs displayed a heterogeneous, bimodal size distribution comprising smaller vesicles (20–60 nm) exosome-like vesicles and larger microvesicle-like populations (200–500 nm) (Liebana-Jordan et al., 2021; Zarnowski et al., 2022). EVs secreted by fluconazole-tolerant isolates appeared to show a relative enrichment in the larger population, which may reflect isolate-specific differences in vesicle biogenesis or cargo packaging (Fu et al., 2025; Johnston et al., 2023; Klimentova and Stulik, 2015). It has been noted that larger EVs are generally associated with plasma membrane budding (microvesicles), whereas the smaller population corresponds to exosome-like vesicles originating from multivesicular bodies (Rizzo et al., 2020). Because EVs were analyzed as naturally released mixtures, the observed immune responses likely reflect the combined activity of multiple EV subpopulations. Both small and large fungal EVs carry immunostimulatory cargo (Ellis and Kuehn, 2010; Joffe et al., 2016), supporting their collective contribution to epithelial immune activation. Future fractionation-based studies will be required to assign size-specific immunological functions.

Extracellular vesicles from both tolerant and susceptible isolates showed robust association with FaDu epithelial cells, suggesting that differences in immune activation are unlikely to be explained by differences in EV association alone. Although EVs are generally reported to undergo cellular internalization via endocytic pathways (Stotz et al., 2022), the fluorescence-based approach used here does not allow definitive discrimination between surface-bound and internalized vesicles. Therefore, the observed signal likely represents a combination of both populations. EV-associated fungal cell wall components, including *β*-glucans, mannoproteins, lipids, secreted proteases, and RNA species are known to engage epithelial pattern-recognition receptors (PRRs), such as TLR2, TLR4, and Dectin-1, leading to activation of NF-κB and MAPK signaling pathways (Ellis and Kuehn, 2010; Nenciarini and Cavalieri, 2023; Patin et al., 2019). In addition to surface recognition, internalized EV cargo may further modulate epithelial responses through endosomal or cytosolic sensing pathways. Thus, epithelial activation likely reflects coordinated contributions from both membrane-associated and intracellular recognition mechanisms, although their relative contributions remain to be defined. Importantly, the involvement of specific signaling pathways (e.g., NF-κB and MAPK) is inferred from prior literature and was not directly investigated in this study; therefore, these mechanisms should be considered conceptual and hypothesis-generating and not experimentally validated in this study. Future studies incorporating higher-resolution imaging, co-localization with endosomal markers (e.g., early endosome antigen 1 [EEA1] and lysosomal-associated membrane protein 1 [LAMP1]), together with receptor-specific perturbation approaches, will be necessary to definitively resolve EV internalization and intracellular trafficking dynamics (Cook et al., 2004; Olson et al., 2023).

Barrier integrity assays further revealed that EVs induced modest and transient reductions in transepithelial electrical resistance without cytotoxicity, consistent with reversible modulation of epithelial tight-junction organization rather than structural damage. Such controlled permeability changes are characteristic of PRR-dependent epithelial immune activation and may facilitate immune surveillance while preserving overall barrier integrity (Hu et al., 2013; McLaughlin et al., 2022). These findings further support the interpretation that tolerant-isolate EVs are associated with enhanced immune signaling without compromising epithelial viability.

The heightened immunostimulatory activity of tolerant-isolate EVs may reflect differences in cargo composition, including enrichment of fungal cell wall components and other bioactive molecules capable of more effectively engaging epithelial PRRs (Joffe et al., 2016; Kozlowska et al., 2025). This enhanced signaling is associated with increased cytokine production, selective induction of antimicrobial peptides, and elevated nitric oxide release-responses that are consistent with, but not definitive evidence of, activation of NF-κB and MAPK pathways in epithelial cells (Feller et al., 2014; Qin et al., 2016; Schaller et al., 2002; Steele and Fidel, 2002). Supporting the broader relevance of these observations, EVs from clinical *C. albicans* isolates have also been shown to induce strong inflammatory responses in macrophages, including TNF-*α*, IL-10, IL-12p40, TGF-*β* and induction of iNOS (Vargas et al., 2015), suggesting that EV-mediated immune modulation extends across multiple host cell types. Importantly, the specific molecular composition of EV cargo responsible for the observed immunomodulatory differences was not directly characterized in this study; therefore, these functional effects cannot be attributed to defined proteomic, transcriptomic, or lipid components and should be interpreted as hypothesis-generating.

Although epithelial immune activation is traditionally associated with fungal clearance, sustained or dysregulated inflammatory responses have been proposed to contribute to tissue damage and persistent infection. In the present study, EV-mediated induction of cytokines, antimicrobial peptides, and nitric oxide indicates robust epithelial activation; however, the functional consequences of this response in terms of fungal clearance or persistence were not directly assessed. It is therefore possible that EVs from fluconazole-tolerant isolates may contribute to mucosal persistence through immune modulation rather than enhanced invasion, although this remains to be experimentally validated. This interpretation is consistent with clinical observations of recurrent candidiasis and requires further experimental validation. Because EVs were isolated under drug-free conditions, the observed differences most likely reflect tolerance-associated traits rather than acute stress responses, although their impact under antifungal pressure *in vivo* remains to be determined. In a complex mucosal environment, such as during oropharyngeal candidiasis, EV-mediated epithelial activation may interact with immune cell recruitment, microbiota, and tissue architecture (Liu and Hu, 2023; Margutti et al., 2024) therefore, the overall impact of these responses on fungal clearance or persistence cannot be inferred from the present *in vitro* model alone.

Several limitations of this study should be acknowledged. The number of clinical isolates included was limited; therefore, the findings should be interpreted as phenotype-associated observations within a limited number of clinical isolates and not generalized to all fluconazole-tolerant or -susceptible *C. albicans* populations. Although consistent responses were observed across independent biological replicates and multiple functional readouts, these findings are based on a small set of isolates and should be considered preliminary and hypothesis-generating. Validation across larger and more diverse isolate collections will be required to confirm the generalizability of these observations.

Extracellular vesicles characterization in this study relied primarily on DLS and total protein content, without orthogonal structural validation, direct particle quantification, or detailed cargo-level analysis. Protein content was used as a proxy for EV abundance; however, this approach does not fully account for potential differences in vesicle number or cargo composition across isolates. Therefore, additional techniques such as transmission electron microscopy (TEM), nanoparticle tracking analysis (NTA), or cryo-electron microscopy (cryo-EM), as well as proteomic or transcriptomic profiling would be required to provide more comprehensive structural and molecular characterization of EV populations. Furthermore, all experiments were conducted using a single oral epithelial cell line (FaDu), which, while providing a reproducible and well-established *in vitro* model for studying epithelial responses to microbial stimuli (Gil-Bona et al., 2015b; Martinez-Lopez et al., 2004), may not fully recapitulate the physiological characteristics and cellular complexity of primary oral epithelial tissues or the oral mucosal environment. Accordingly, the generalizability of these findings is limited and future studies using primary epithelial cells and more physiologically relevant models will be important to validate and extend these observations.

**Figure 9:**
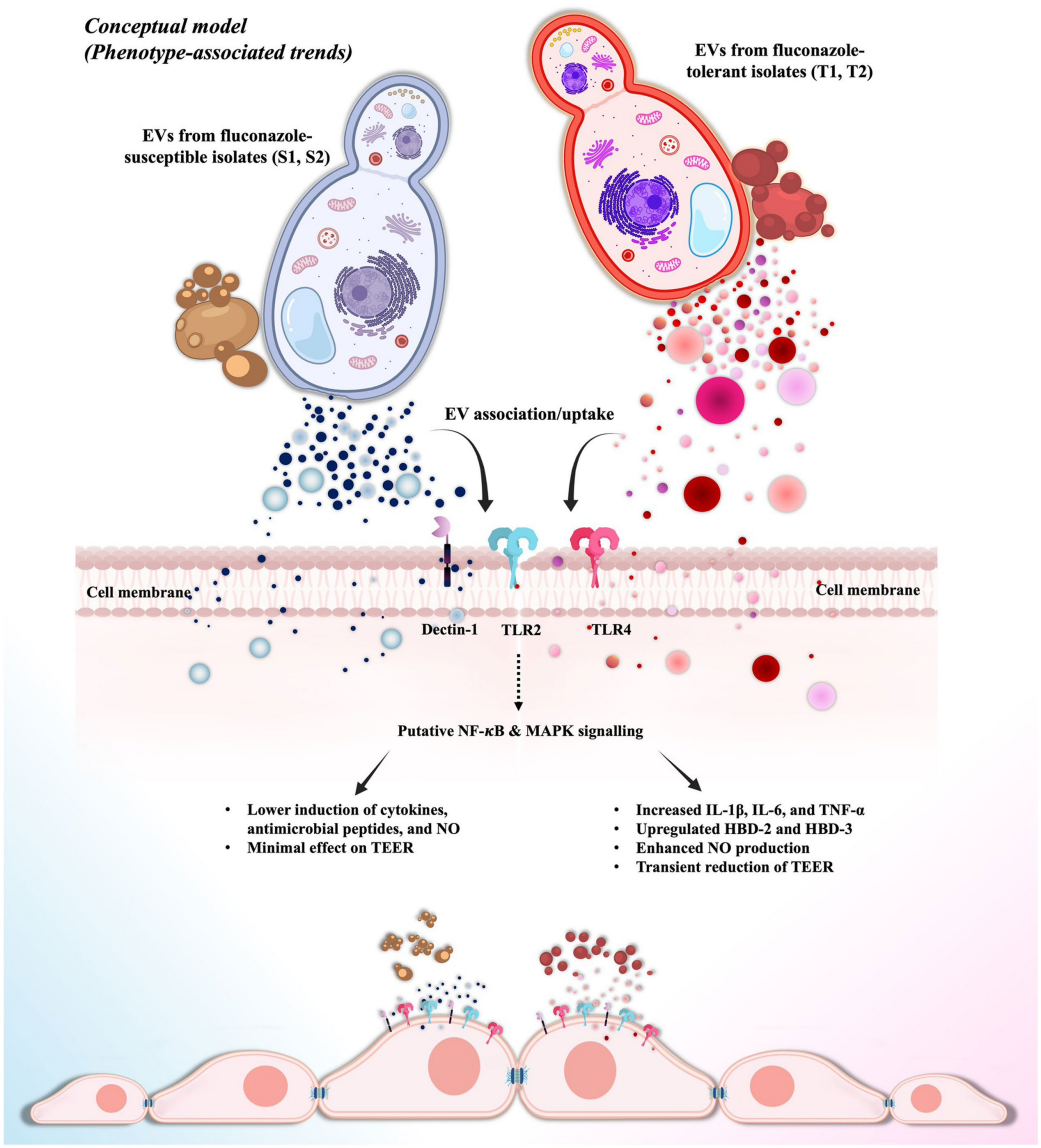
Hypothesis-generating working model of EV-mediated epithelial immune activation. EVs released by fluconazole-tolerant C. albicans isolates (T1, T2) are associated with stronger epithelial immune responses compared with EVs from fluconazole-susceptible isolates (S1, S2), within this experimental system. Based on the present findings and supporting literature, EV-associated components, including fungal cell wall molecules, proteins, lipids, and RNAs, are proposed to interact with epithelial pattern-recognition receptors (PRRs) and may contribute to the activation of downstream signaling pathways such as NF-κB and MAPK. This is associated with higher expression of proinflammatory cytokines (IL-1β, IL-6, TNF-α), antimicrobial peptides (hBD-2, hBD-3), and nitric oxide, accompanied by modest and transient changes in epithelial barrier function. This model is conceptual and intended to illustrate phenotype-associated trends observed in a limited number of clinical isolates rather than to represent a definitive or universally applicable mechanism. Direct molecular validation of PRR engagement and downstream signaling was not performed and remains an important direction for future studies.

While our findings demonstrate differences in EV-mediated epithelial immune activation under *in vitro* conditions, these observations are based on functional assays, and direct associations with disease severity, fungal burden, or persistence cannot be established. Additionally, EVs were analyzed as unfractionated populations to reflect naturally released vesicle mixtures; however, distinct EV subpopulations may differ in composition and immunomodulatory activity. Future studies incorporating larger isolated cohorts, EV subpopulation fractionation, and multi-omics profiling, as well as relevant *in vivo* models, such as murine models of oropharyngeal candidiasis (OPC), will be necessary to validate and extend these findings. Within these limitations, our results suggest that fluconazole tolerance is associated with qualitative differences in EV-mediated epithelial immune activation within this experimental system. Based on these observations, we propose a conceptual, hypothesis-generating model of EV-mediated epithelial immune activation, as illustrated in Figure 9, which reflects phenotype-associated trends observed in a limited number of clinical isolates rather than a definitive or universally applicable mechanism.

## 5 Conclusion

In conclusion, our findings indicate that fluconazole tolerance is associated with distinct extracellular vesicle (EV)-mediated modulation of epithelial innate immune responses within this experimental system. EVs derived from the tested tolerant isolates exhibited enhanced immunostimulatory capacity, independent of cytotoxicity, barrier disruption, or differences in EV association. These results highlight the potential functional relevance of antifungal tolerance in shaping host-pathogen interactions at the epithelial interface. Collectively, this study supports a hypothesis-generating framework in which EVs may act as mediators linking antifungal tolerance to epithelial immune conditioning in *C. albicans*.

## Data Availability

The original contributions presented in the study are included in the article/Supplementary material, further inquiries can be directed to the corresponding author.
