# Supplementary material for: Fluconazole tolerance is associated with altered extracellular vesicle-mediated epithelial immune responses in Candida albicans
Source: Front Microbiol. 2026 Apr 28;17:1807232. doi: 10.3389/fmicb.2026.1807232 (PMC13161164; doi:10.3389/fmicb.2026.1807232)
Supplement: Supplementary file 1 [file Table_1.DOCX]

Supplementary Material

# Supplementary Table S1. Primer sequences used for real-time PCR.

| **Gene** | **Sequence (5’->3’)** | **Reference** |
| --- | --- | --- |
| *IL-1β* | F: AGCTGGAGAGTGTAGATCCCAA | (Toth *et al.*, 2017) |
|  | R: GGGAACTGGGCAGACTCAAA |  |
| *IL-6* | F: TGCAATAACCACCCCTGACC | (X. Zhang *et al.*, 2016) |
|  | R: GTGCCCATGCTACATTTGCC |  |
| *TNF-α* | F: CCCAGGGACCTCTCTCTAATC | (Yi *et al.*, 2017) |
|  | R: ATGGGCTACAGGCTTGTCACT |  |
| *TGF-β* | F: GCCCTGGACACCAACTATTGCT | (Takizawa *et al.*, 2001) |
|  | R: AGGCTCCAAATGTAGGGGCAGG |  |
| *IL-8* | F: AAGGAACCATCTCACTGTGTGTAAAC | (Harrison *et al.*, 2005) |
|  | R: ATCAGGAAGGCTGCCAAGAG |  |
| *IL-10* | F: GTGATGCCCCAAGCTGAGA | (Sapan *et al.*, 2017) |
|  | R: CACGGCCTTGCTCTTGTTTT |  |
| *IL-12* | F: GCGGAGCTGCTACACTCTCT | (W. Zhang *et al.*, 2016) |
|  | R: GGTGGGTCAGGTTTGATGAT |  |
| *IL-17* | F: ATCTCCACCGCAATGAGGAC | (Moutia *et al.*, 2016) |
|  | R: GTGGACAATCGGGGTGACAC |  |
| *iNOS* | F: TCCAAGGTATCCTGGAGCGA | (W. Zhang *et al.*, 2016) |
|  | R: CAGGGACGGGAACTCCTCTA |  |
| *HBD-1* | F: CAGGTGGTAACTTTCTCACAGG | (Bonamy *et al.*, 2018) |
|  | R: AATAGAGACATTGCCCTCCACT |  |
| *HBD-2* | F: GCCATGAGGGTCTTGTATCTC |  |
|  | R: TTAAGGCAGGTAACAGGATCG |  |
| *HBD-3* | F: TTTGGTGCCTGTTCCAGGTCAT |  |
|  | R: GCCGCCTCTGACTCTGCAATAATA |  |
| *PR-39-derived cathelicidin* | F: ACCCATCCATTCACTCAC | (Wu *et al.*, 1999) |
|  | R: AGCCACAACAATAAGATCC |  |
| *β-actin* | F: ATTGCCGACAGGATGCAGAA | (Maess *et al.*, 2010) |
|  | R: GCTGATCCACATCTGCTGGAA |  |

*β-actin* was used as the internal reference gene for normalization in all qRT-PCR analyses.

**Supplementary Table S2.** Raw qRT-PCR expression data (ΔΔCt) of cytokine genes in FaDu cells exposed to EVs from fluconazole-tolerant and -susceptible *C. albicans* isolates.

| **Gene** | **Condition** | **Replicate 1** | **Replicate 2** | **Replicate 3** | **Mean** | **SD** |
| --- | --- | --- | --- | --- | --- | --- |
| *IL-1β* | EV released by T1 | 1.964 | 2.282 | 2.774 | 2.340 | 0.408 |
|  | EV released by T2 | 3.216 | 3.142 | 2.847 | 3.068 | 0.195 |
|  | EV released by S1 | 2.013 | 1.423 | 1.364 | 1.600 | 0.359 |
|  | EV released by S2 | 1.851 | 1.975 | 1.102 | 1.643 | 0.472 |
| *IL-6* | EV released by T1 | 3.105 | 4.218 | 3.953 | 3.759 | 0.581 |
|  | EV released by T2 | 4.148 | 2.851 | 3.943 | 3.647 | 0.697 |
|  | EV released by S1 | 2.154 | 1.682 | 1.640 | 1.825 | 0.285 |
|  | EV released by S2 | 1.123 | 2.081 | 1.642 | 1.615 | 0.480 |
| *TNF-α* | EV released by T1 | 4.529 | 3.744 | 2.983 | 3.752 | 0.773 |
|  | EV released by T2 | 5.327 | 6.144 | 6.018 | 5.830 | 0.440 |
|  | EV released by S1 | 3.417 | 2.984 | 3.060 | 3.154 | 0.231 |
|  | EV released by S2 | 1.655 | 1.993 | 2.454 | 2.034 | 0.401 |
| *TGF-β* | EV released by T1 | 1.127 | 0.894 | 0.981 | 1.001 | 0.118 |
|  | EV released by T2 | 1.564 | 1.326 | 1.036 | 1.309 | 0.264 |
|  | EV released by S1 | 2.417 | 1.651 | 1.743 | 1.937 | 0.418 |
|  | EV released by S2 | 2.063 | 1.647 | 1.742 | 1.817 | 0.218 |
| *IL-8* | EV released by T1 | 1.264 | 2.147 | 2.174 | 1.862 | 0.518 |
|  | EV released by T2 | 2.167 | 2.653 | 2.114 | 2.311 | 0.297 |
|  | EV released by S1 | 1.962 | 1.342 | 2.410 | 1.905 | 0.536 |
|  | EV released by S2 | 2.063 | 1.642 | 2.741 | 2.149 | 0.554 |
| *IL-10* | EV released by T1 | 0.891 | 1.310 | 2.148 | 1.450 | 0.640 |
|  | EV released by T2 | 0.642 | 0.523 | 0.638 | 0.601 | 0.068 |
|  | EV released by S1 | 1.236 | 0.684 | 1.320 | 1.080 | 0.346 |
|  | EV released by S2 | 2.174 | 2.451 | 1.362 | 1.996 | 0.566 |
| *IL-12* | EV released by T1 | 1.237 | 1.147 | 0.784 | 1.056 | 0.240 |
|  | EV released by T2 | 1.045 | 1.583 | 1.476 | 1.368 | 0.285 |
|  | EV released by S1 | 2.012 | 1.620 | 1.646 | 1.759 | 0.219 |
|  | EV released by S2 | 1.142 | 0.784 | 0.985 | 0.970 | 0.179 |
| *IL-17* | EV released by T1 | 1.172 | 1.961 | 1.117 | 1.417 | 0.472 |
|  | EV released by T2 | 1.782 | 1.356 | 1.123 | 1.420 | 0.334 |
|  | EV released by S1 | 1.85 | 1.081 | 1.678 | 1.536 | 0.404 |
|  | EV released by S2 | 1.643 | 2.306 | 1.577 | 1.842 | 0.403 |
| *iNOS* | EV released by T1 | 1.794 | 1.122 | 1.080 | 1.332 | 0.401 |
|  | EV released by T2 | 1.662 | 1.744 | 2.06 | 1.822 | 0.210 |
|  | EV released by S1 | 1.641 | 2.451 | 1.362 | 1.818 | 0.566 |
|  | EV released by S2 | 2.115 | 1.745 | 1.846 | 1.902 | 0.191 |

ΔΔCt values were calculated using β-actin as the internal reference gene and normalized to untreated control cells. Data represent three independent biological experiments. For group-level comparisons, isolate means were grouped as fluconazole-tolerant (T1 + T2) and fluconazole-susceptible (S1 + S2), and analyzed using one-way ANOVA followed by Tukey’s multiple comparison test. The results revealed significantly higher expression of *IL-1β* (*p* = 0.024), *IL-6* (*p* = 0.006), and *TNF-α* (*p* = 0.018) in FaDu cells treated with EVs derived from fluconazole-tolerant isolates. No significant group differences were observed for *TGF-β, IL-8, IL-10, IL-12, IL-17*, or *iNOS* (*p* > 0.05).

**Supplementary Table S3.** Raw qRT-PCR expression data (ΔΔCt) of antimicrobial peptides in FaDu cells exposed to EVs from fluconazole-tolerant and -susceptible *C. albicans* isolates.

| **Gene** | **Condition** | **Replicate 1** | **Replicate 2** | **Replicate 3** | **Mean** | **SD** |
| --- | --- | --- | --- | --- | --- | --- |
| *HBD-1* | EV released by T1 | 1.325 | 2.280 | 1.876 | 1.827 | 0.479 |
|  | EV released by T2 | 3.120 | 2.050 | 2.630 | 2.600 | 0.536 |
|  | EV released by S1 | 2.871 | 2.145 | 1.989 | 2.335 | 0.471 |
|  | EV released by S2 | 1.874 | 2.314 | 2.364 | 2.184 | 0.270 |
| *HBD-2* | EV released by T1 | 5.103 | 4.142 | 5.369 | 4.871 | 0.645 |
|  | EV released by T2 | 5.970 | 7.456 | 6.149 | 6.525 | 0.811 |
|  | EV released by S1 | 1.358 | 1.133 | 2.145 | 1.545 | 0.531 |
|  | EV released by S2 | 3.687 | 2.478 | 4.016 | 3.394 | 0.810 |
| *HBD-3* | EV released by T1 | 7.148 | 7.337 | 6.144 | 6.876 | 0.641 |
|  | EV released by T2 | 9.149 | 8.145 | 7.470 | 8.255 | 0.845 |
|  | EV released by S1 | 1.325 | 2.018 | 2.647 | 1.997 | 0.661 |
|  | EV released by S2 | 3.467 | 3.548 | 2.648 | 3.221 | 0.498 |
| *PR-39* | EV released by T1 | 2.795 | 2.197 | 2.648 | 2.547 | 0.312 |
|  | EV released by T2 | 3.254 | 4.684 | 3.170 | 3.703 | 0.851 |
|  | EV released by S1 | 3.149 | 3.500 | 3.987 | 3.545 | 0.421 |
|  | EV released by S2 | 3.364 | 2.780 | 3.145 | 3.096 | 0.295 |

Group-level statistical analysis (tolerant; T1 + T2 vs susceptible; S1 + S2) using one-way ANOVA with Tukey’s multiple comparison test revealed significantly higher expression of *HBD-2* (*p* = 0.01) and *HBD-3* (*p* < 0.001) in cells treated with EVs from tolerant isolates, whereas no significant differences were observed for HBD-1 or PR-39 (*p* > 0.05).

**Supplementary Table S4.** Nitric oxide production by FaDu cells after exposure to EVs from fluconazole-tolerant and -susceptible *C. albicans* isolates.

| **Condition** | | **Replicate 1 (µM)** | **Replicate 2 (µM)** | **Replicate 3 (µM)** | **Mean** | **SD** |
| --- | --- | --- | --- | --- | --- | --- |
| 3 h | EV released by T1 | 30.25 | 28.36 | 27.55 | 28.72 | 1.39 |
|  | EV released by T2 | 35.25 | 33.15 | 31.67 | 33.36 | 1.80 |
|  | EV released by S1 | 18.35 | 18.39 | 20.11 | 18.95 | 1.00 |
|  | EV released by S2 | 21.32 | 23.37 | 28.60 | 24.43 | 3.75 |
|  | Untreated control | 11.78 | 15.36 | 12.94 | 13.36 | 1.86 |
| 6 h | EV released by T1 | 35.57 | 33.54 | 37.69 | 35.60 | 2.08 |
|  | EV released by T2 | 41.30 | 38.74 | 33.50 | 37.85 | 3.98 |
|  | EV released by S1 | 20.35 | 22.67 | 25.30 | 22.77 | 2.48 |
|  | EV released by S2 | 25.69 | 29.48 | 30.27 | 28.48 | 2.45 |
|  | Untreated control | 13.35 | 15.54 | 15.32 | 14.74 | 1.21 |

Nitric oxide concentrations were determined using the Griess assay. Group-level statistical analysis (tolerant; T1 + T2 vs susceptible; S1 + S2) using one-way ANOVA with Tukey’s multiple comparison test demonstrated significantly higher NO production in FaDu cells treated with EVs from fluconazole-tolerant isolates compared with susceptible isolates at 3 h (*p* = 0.0008) and 6 h (*p* = 0.0002).

**References**

Bonamy, C., Sechet, E., Amiot, A., Alam, A., Mourez, M., Fraisse, L., Sansonetti, P. J., & Sperandio, B. (2018). Expression of the human antimicrobial peptide beta-defensin-1 is repressed by the EGFR-ERK-MYC axis in colonic epithelial cells. Sci Rep, 8(1): 18043.

Harrison, L. M., van den Hoogen, C., van Haaften, W. C., & Tesh, V. L. (2005). Chemokine expression in the monocytic cell line THP-1 in response to purified shiga toxin 1 and/or lipopolysaccharides. Infect Immun, 73(1): 403–412.

Maess, M. B., Sendelbach, S., & Lorkowski, S. (2010). Selection of reliable reference genes during THP-1 monocyte differentiation into macrophages. BMC Mol Biol, 11: 90.

Moutia, M., Seghrouchni, F., Abouelazz, O., Elouaddari, A., Al Jahid, A., Elhou, A., Nadifi, S., Jamal Eddine, J., Habti, N., & Badou, A. (2016). *Allium sativum* L. regulates *in vitro* IL-17 gene expression in human peripheral blood mononuclear cells. BMC Complement Altern Med, 16(1): 377.

Sapan, H. B., Paturusi, I., Islam, A. A., Yusuf, I., Patellongi, I., Massi, M. N., Pusponegoro, A. D., Arief, S. K., Labeda, I., Rendy, L., & Hatta, M. (2017). Interleukin-6 and interleukin-10 plasma levels and mRNA expression in polytrauma patients. Chin J Traumatol, 20(6): 318–322.

Takizawa, H., Tanaka, M., Takami, K., Ohtoshi, T., Ito, K., Satoh, M., Okada, Y., Yamasawa, F., Nakahara, K., & Umeda, A. (2001). Increased expression of transforming growth factor-beta1 in small airway epithelium from tobacco smokers and patients with chronic obstructive pulmonary disease (COPD). Am J Respir Crit Care Med, 163(6): 1476–1483.

Toth, E. J., Boros, E., Hoffmann, A., Szebenyi, C., Homa, M., Nagy, G., Vagvolgyi, C., Nagy, I., & Papp, T. (2017). Interaction of THP-1 monocytes with conidia and hyphae of different *Curvularia* strains. Front Immunol, 8: 1369.

Wu, H., Zhang, G., Ross, C. R., & Blecha, F. (1999). Cathelicidin gene expression in porcine tissues: roles in ontogeny and tissue specificity. Infect Immun, 67(1): 439–442.

Yi, L., Shen, H., Zhao, M., Shao, P., Liu, C., Cui, J., Wang, J., Wang, C., Guo, N., Kang, L., Lv, P., Xing, L., & Zhang, X. (2017). Inflammation-mediated SOD-2 upregulation contributes to epithelial-mesenchymal transition and migration of tumor cells in aflatoxin G(1)-induced lung adenocarcinoma. Sci Rep, 7(1): 7953.

Zhang, W., Chen, L., Ma, K., Zhao, Y., Liu, X., Wang, Y., Liu, M., Liang, S., Zhu, H., & Xu, N. (2016). Polarization of macrophages in the tumor microenvironment is influenced by EGFR signaling within colon cancer cells. Oncotarget, 7(46): 75366–75378.

Zhang, X., Li, S., Li, M., Huang, H., Li, J., & Zhou, C. (2016). Hypoxia-inducible factor-1alpha mediates the toll-like receptor 4 signaling pathway leading to anti-tumor effects in human hepatocellular carcinoma cells under hypoxic conditions. Oncol Lett, 12(2): 1034–1040.
